# Non-Thermal Plasma-Mediated Redox Signaling and Microbiome Interactions for Abiotic Stress Adaptation: Molecular Insights and Future Prospects for Sustainable Agriculture

**DOI:** 10.3390/ijms27177656

**Published:** 2026-08-26

**Authors:** Rida Javed, Guangyao Ji, Qi Sun, Feng Huang

**Affiliations:** 1 College of Information and Electronic Engineering, China Agricultural University, Beijing 100083, China; ridajaved.uaf@cau.edu.cn; 2 College of Science, China Agricultural University, Beijing 100083, China; jiguangyao@cau.edu.cn (G.J.); sqi0715@163.com (Q.S.)

**Keywords:** abiotic stress, plant growth-promoting bacteria, non-thermal plasma, reactive oxygen and nitrogen species, antioxidants, stress tolerance mechanism

## Abstract

Crop production is continually exposed to a wide range of abiotic stresses that negatively affect growth and yield, posing a severe threat to global food security. Plant growth-promoting bacteria (PGPB) promote nutrient assimilation, activate antioxidant enzymes, and stimulate phytohormone production to mitigate abiotic stress. However, the effective application of PGPB in the field depends on host colonization, soil specificity, and susceptibility to competitive microbial communities. Recently, non-thermal plasma (NTP) has emerged as a revolutionary tool for sustainable agriculture, making it a priority to develop efficient, low-cost, and eco-friendly strategies to enhance seed vitality and manage abiotic stress. Plasma-generated reactive oxygen and nitrogen species (RONS) have been shown to mediate intracellular redox homeostasis and the antioxidant defense signaling network. Furthermore, plasma stimulates MAPK cascades and stress-responsive genes such as *LEA1*, *SnRK2*, *P5C*, and the *SOS* pathway, ionic balance, and membrane stability, ultimately supporting plant stress adaptation to drought, salinity, and heavy metals. Plasma-induced RONS signaling activates PGPB functional traits such as root colonization, biofilm formation, nutrient mobilization, and plant growth-promoting activities. However, the molecular mechanisms underlying NTP-PGPB microbial multiple stress adaptation and the long-term ecological stability and biosafety of microbial communities remain inadequately resolved. Consequently, future integration of multi-omics approaches, synthetic microbial communities, and field-scale validation is required to explore the mechanistic advances of plasma-modulated microbiome interactions to enable agricultural applications.

## 1. Introduction

Sustainable agriculture provides an integrated approach to address critical challenges in modern farming systems, including environmental degradation and economic instability. The excessive reliance on agrochemicals such as fertilizers and pesticides has exacerbated soil and water pollution, accelerated the development of pest and weed resistance, and substantially increased agriculture-related greenhouse gas emissions [1]. Furthermore, climate change has increased agricultural stress due to extreme fluctuations and severity of weather events, such as droughts, salinity, and heavy metal stress, which directly affect soil health and crop production [2]. Abiotic stressors affect plant physiological responses, altering gene expression in metabolic pathways, changing growth dynamics, and ultimately leading to significant decreases in crop yield [3]. Moreover, drought is a crucial abiotic stressor that affects food security and crop yields worldwide. The area affected by drought stress has increased by almost 1.2 × 10^2^ km^2^. Drought stress severely affects physiological and biochemical functions in plants, including water potential and turgor pressure, and reduces soil nutrient availability, increases oxidative stress, inhibits metabolic activity, and lowers yields of major crops such as wheat, rice, maize, and barley [4]. Soil salinity affects around 18–21% of cultivated land worldwide, which is nearly 450,000 km^2^, and causes the loss of 2500–5000 km^2^ of productive agricultural land each year. Despite the growing global population, the effective reclamation and implementation of salt-affected soils is critical for maintaining global food security [5].

High salt levels cause ionic imbalances and nutritional deficiencies by interfering with nutrient absorption [6]. In addition to other stresses, high concentrations of toxic metals (Mn, Pb, Cd, and Cr) in soil, due to rapid industrialization and population growth, severely restrict crop productivity [7]. The agricultural sector requires simultaneous improvements in crop yield and output quality to overcome climate-induced stress [8]. Moreover, plant microbiome engineering provides an effective approach to enhancing growth and stress resistance by improving hormone production, nutrient availability, antimicrobial activity, and soil productivity [9]. 

Plant growth-promoting bacteria (PGPB) enhance plant growth, development, and resistance to heavy metal stress. PGPB synthesize siderophores to chelate iron and minerals, thereby solubilizing them in plant roots [10]. Moreover, the growing world population puts food security at risk and increases reliance on chemical fertilizers, which harm the environment and human health. Environmental stresses further decrease crop yields. PGPB support organic farming, thereby improving crop sustainability, productivity, and quality [11]. Most PGPB groups include *Erwinia*, *Bacillus*, *Enterobacter*, and *Pseudomonas*. PGPB converts 1-aminocyclopropane-1-carboxylate (ACC) into ammonia, enhancing nutrient uptake and reducing ethylene-mediated responses, and generate indole-3-acetic acid to regulate auxin signaling [12]. PGPB can induce stress tolerance in plants, and their practical application under abiotic stress conditions remains constrained by several critical limitations. PGPB are crucial for root colonization, survival, and consistent functional expression, but osmotic imbalance, nutrient limitation, and competition with indigenous microbiota limit their performance. Moreover, the efficacy of PGPB also depends on soil characteristics and stress specificity, leading to poor reproducibility under field conditions. 

Recently, plasma agriculture has emerged as a promising approach that activates plant- and microbial stress-responsive pathways. The integration of plasma agricultural technologies has opened new avenues for improving crop productivity while addressing food security and environmental sustainability [13]. Plasma generation has unique methods and can be modified to meet specific agricultural requirements. Plasma-treated water (PTW) is produced by exposing water to reactive oxygen and nitrogen species (RONS) containing discharges [14]. PTW can improve microbial redox homeostasis, membrane permeability, biofilm formation, and antioxidant enzyme activity. However, PTW produces RONS that act as signaling molecules, boosting seed germination, root exudation, and rhizosphere microbial recruitment while maintaining ecological safety at optimal levels. Plasma can influence plant microbiomes and increase plant resistance. Optimizing plasma will be essential for developing novel treatment options to advance plant research. Recent studies have shown that plasma treatment significantly affects plasmidome diversity, gene expression patterns, and metabolite synthesis [15]. 

This review illustrates how plasma technologies can improve plant stress resilience and highlights applications of research on plant microbiome stimulation. Despite significant advances in plant microbiome research and plasma agriculture, the mechanistic understanding of how non-thermal plasma modulates plant-microbe interactions under abiotic stress remains unexplored. In particular, integration of plasma-induced physicochemical changes with microbial functionality and host plant signaling pathways remains elusive. Addressing this gap is essential for developing reliable, field-applicable plasma-based strategies for sustainable agriculture. 

The main objective of this review is to (i) evaluate the impact of abiotic stress on crop productivity; (ii) critically analyze the role and limitations of plant growth-promoting bacteria in stress mitigation; (iii) understand the mechanism of plasma technology to enhance plant stress resilience; and (iv) synergistically integrate plasma-mediated plant-microbe interactions, highlighting how plasma technology enhances microbial functionality and activates plant stress-responsive pathways to promote sustainable stress resilience. Furthermore, plasma treatments may also reduce reliance on chemical fertilizers while increasing the nutrient availability and physicochemical characteristics of soil. Plasma technology has shown potential in agriculture, although its application in crop plant stress management has not been thoroughly investigated. This review summarizes recent developments in plasma technology, focusing on the molecular mechanisms of abiotic stress resilience, and explores the potential of plasma-mediated plant-microbe interactions.

## 2. Effect of Abiotic Stresses on Plant Yield and Productivity

Climate change induces multiple abiotic stresses that decrease agricultural yields and productivity and deplete natural resources [16]. The main abiotic stresses, including drought, salinity, and heavy metal contamination, significantly reduce crop productivity and threaten global food security [17]. Drought and salinity are among the most severe abiotic stresses that disrupt plant growth and yield by promoting oxidative stress, osmotic imbalance, and impaired nutrient acquisition [18]. Drought stress restricts gas exchange and diminishes photosynthesis, thereby reducing leaf area and cellular water potential, ultimately constraining plant growth and enhancing ROS production [19]. Apart from that, the excessive accumulation of sodium (Na^+^) and chloride (Cl^−^) ions in the cytosol disrupts cellular function and significantly impairs plant yield and development [20]. Moreover, heavy metal stress disrupts cellular structures and alters protein function by generating free radicals and excessive accumulation of ROS in plants, thereby compromising membrane integrity. Heavy metals also pose serious risks to human health, including metabolic, cardiovascular, and carcinogenic effects, and they accumulate through the food chain [21]. 

**Figure 1:**
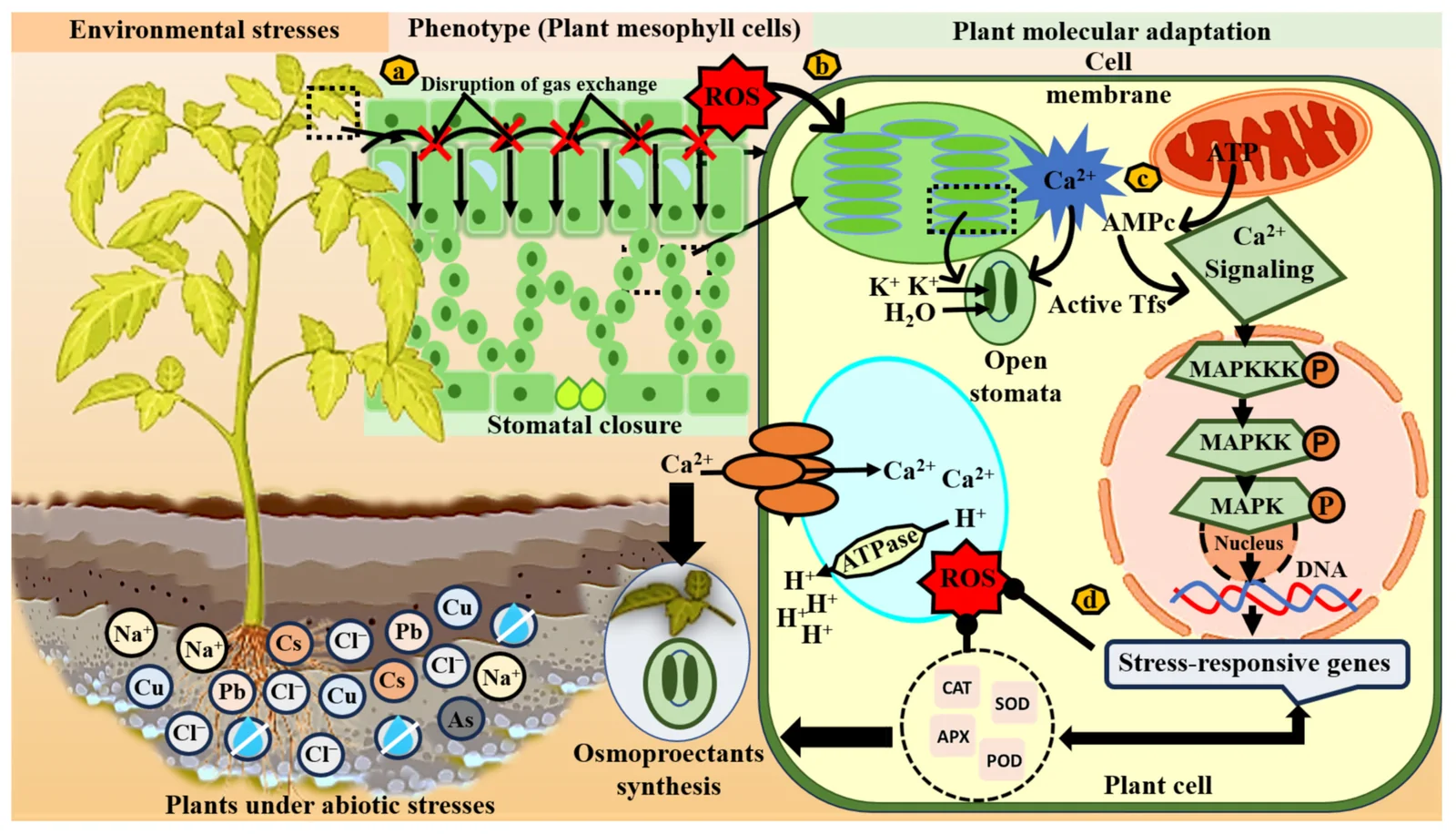
Molecular mechanism of abiotic stressors in plant integration and adaptation: (**a**) stress perception by plasma membrane receptors; (**b**) the Ca^2+^ and ROS signaling pathways that occur in the endoplasmic reticulum and peroxisomes; (**c**) the cytosolic Ca^2+^ influx-induced activation of protein kinases, including calcium-dependent protein kinases (CDPKs) and MAPK (Mitogen Activated Protein Kinase) cascades, which may activate phytohormone biosynthesis; (**d**) finally, the transcriptional modulation of the expression of multiple genes expression in the nucleus leads to plant adaptation. Figure(s) partially created in BioRender. Javed, R. (2026) https://BioRender.com/cqkqald.

Abiotic stressors, for instance, drought, salinity, and heavy metals, are responsible for prevalent cellular disorders in plants, including osmotic stress, membrane dysfunction, excessive ROS formation, and protein destabilization. Stress tolerance in plants depends on a tightly integrated defense system that operates at the cellular, physiological, and morphological levels [22]. The molecular sensing mechanism in plants under abiotic stress maintains cellular homeostasis, activates antioxidant defenses, and promotes the accumulation of suitable osmolytes (Figure 1).

However, plant defense responses are mediated by a complex signaling network composed of secondary chemical messengers such as ROS, calcium ions (Ca^2+^), nitric oxide (NO), hydrogen sulfide (H_2_S), polyamines, and phytochromes, as well as stress-responsive phytohormones such as ethylene and abscisic acid (ABA). Protein kinases and redox-regulatory proteins mediate signal perception and transmission by triggering stress-responsive transcription factors and modulating the expression of downstream genes [22,23]. Plants use membrane-localized receptors to detect environmental stressors and initiate signal transduction. The sensation of stress rapidly alters membrane ion fluxes, which causes a brief rise in cytosolic Ca^2+^ levels. Calcium acts as a key second messenger, activating calcium-binding proteins and protein kinases that relay signals to the nucleus. The cellular cascades regulate stress-responsive transcription factors, which ultimately modulate stress tolerance inside the plant cell [24]. However, the stress-response systems are insufficient to prevent cellular damage, particularly because of excessive ROS accumulation; plants initiate stress-induced programmed cell death pathways. Prolonged activation of these responses promotes tissue degeneration and ultimately accelerates plant senescence [25,26].

## 3. Role of PGPB in Mitigating Abiotic Stresses

**Figure 2:**
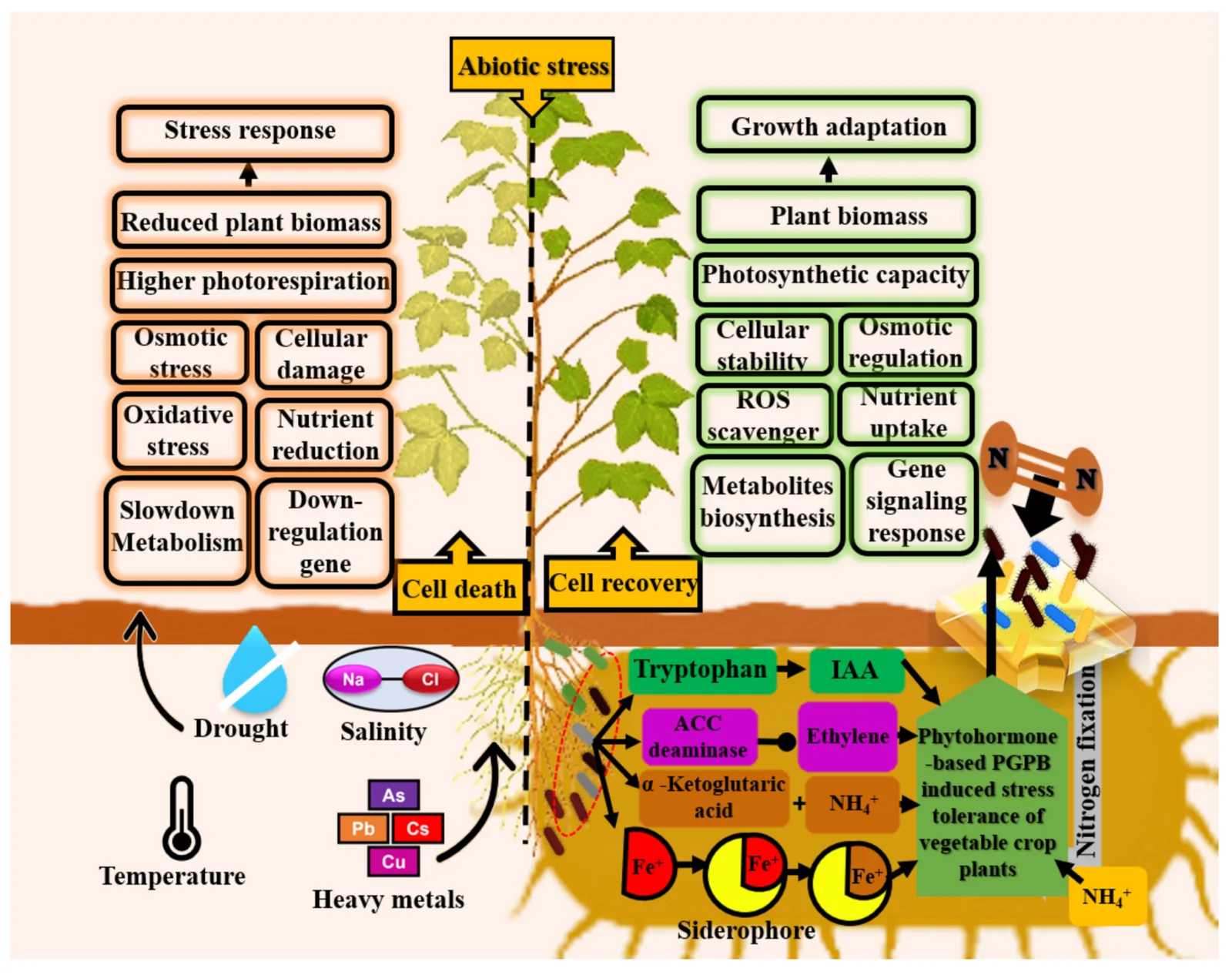
Molecular mechanism of PGPB traits inducing the plant defense system and contributing to plant resilience under abiotic stress. PGPB directly promote plant growth and development through nitrogen fixation, nutrient mobilization, and the production of essential phytohormones. They indirectly influence the synthesis of enzymes and siderophores, thereby improving plant growth and development and increasing the plant’s resistance to external stressors. Figure(s) partially created in BioRender. Javed, R. (2026) https://BioRender.com/jlpcokh.

The plant root system is one of the most susceptible and dynamic ecosystems, supporting a wide variety of microorganisms that play a key role in plant growth and survival. PGPB can reduce plant dependence on external nitrogen inputs by directly fixing and mobilizing nitrogen or indirectly supporting nitrogen fixation through secreted compounds and modifications to the root framework [27]. Most PGPB synthesize plant hormones, including the growth regulator indole-3-acetic acid (IAA), gibberellins, and abscisic acid (ABA). The most common hormone is IAA, which is synthesized by almost 80% of soil microorganisms, especially *Pseudomonas*, *Bacillus*, *Burkholderia*, and *Rhizobium* sp. [3,28]. Moreover, PGPB possess ACC deaminase, which converts plant-produced ACC into ammonia and α-ketobutyrate. This activity decreases ACC availability as a substrate in ethylene biosynthesis, thereby limiting stress-induced overproduction of ethylene. PGPB produce ACC deaminase, as in *Pseudomonas oryzihabitans*, *Bacillus amyloliquefaciens*, and *Burkholderia phytofirmans*, which have been shown to increase root elongation in host plants [29]. Below is the representation of PGPB and the underlying mechanisms by which they improve plant performance under abiotic stress conditions (Figure 2). 

PGPB increases ABA production, thereby suppressing ethylene signaling and controlling plant responses to abiotic stress by altering stomatal conductance. The stress tolerance induced by ABA depends on the plant genotype and the bacterial strain. Several PGPB, such as *Azospirillum brasilense*, *Achromobacter xylosoxidans*, *Bacillus licheniformis*, *B. pumilus*, and *Rhizobium* spp., are producers of ABA during drought stress. ABA signaling induced by PGPB under water-deficit conditions enhances ROS generation and Ca^2+^ channel opening, leading to stomatal closure and reduced water loss [29]. This review addresses the challenges of using PGPB as biopriming agents to increase agricultural productivity in the face of climate change. It focuses on the processes underlying PGPB-mediated abiotic stress tolerance considering recent developments in plant-microbe interactions [3,30].

### 3.1. PGPB as a Strategy to Mitigate Drought Stress

PGPB releases osmolytes that facilitate plant growth under drought stress. Plants maintain cellular osmotic balance with osmolytes, which stimulate stress tolerance [31]. The plant’s roots harbor bacteria that produce ACC deaminase enzymes and regulate ethylene signaling pathways, thereby reducing root dehydration. ACC deaminase activity of *Achromobacter piechaudii* has been reported to enhance the growth of pepper and tomato plants under drought stress [32]. The colonization of PGPB reduced ethylene production under drought stress without significantly altering relative water content. The relative water content of maize increased when *Azospirillum brasilense* was inoculated into the plants, compared to non-inoculated plants [33]. PGPB enhance plant growth and induce tolerance to drought stress. PGPB produce exopolysaccharides that protect plants from dehydration damage and enhance micronutrient uptake during plant growth [34]. 

### 3.2. Role of PGPB Exposed to Salinity Stress

Most studies of PGPB activity under saline stress are conducted in controlled laboratory environments, which are not necessarily realistic simulations of microbial behavior in natural soil. The ACC deaminase, indole-3-acetic acid synthesis, phosphate solubilization, and exopolysaccharide biosynthesis of Bacillus pumilus JPV11 are all notably inhibited with an increase in the concentration of NaCl (0 to 1.2 M) [35]. The PGPB characteristics and viability of Enterobacter, Pseudomonas, Rhizobium, and *Ensifer fredii* were tested in the presence of NaCl (1–5%). The salt-tolerant Enterobacter sp. ECD1 survived at 50% at 5% NaCl, followed by *Pseudomonas* and *Rhizobium* spp., and *E. fredii* had the least tolerance, with rhizobia being the most sensitive. Higher salinity suppressed IAA and siderophore production, in agreement with previous results. PGPB were more salt-tolerant than endophytes, likely due to adaptation at the root surface [36,37]. However, the high salt concentration also affects growth and characteristics, although the extent of the effect varies depending on which PGPB traits are applied. The most preferred PGPB trait depends on the efficiency of plant development, which shows consistent growth and maintains PGPB properties under salinity conditions.

### 3.3. Effect of PGPB Exposed to Heavy Metal Stress

Micronutrients and macronutrients, including potassium, copper, iron, zinc, and phosphorus, cannot move easily in most soils. The solubilization and mobilization of insoluble soil phosphorus are mainly driven by root exudates, organic acids, chelators, and phosphatase enzymes released during plant root development and the related root morphology of microbes [38]. Metal-resistant PGPB mobilize metals via biomethylation, thereby facilitating their volatilization. The process of arsino (As) methylation produces trace amounts of monomethylarsinoic acid and dimethylarsinoic acid (DMA) in soil. Methylated As roots are less readily absorbed compared to inorganic forms. DMA does not precipitate with plant chelators or undergo vacuolar sequestration, thereby enabling efficient translocation. Pseudomonas alcaligenes As (III) S-adenosylmethionine methyltransferase is a protein that catalyzes methylation and detoxification, which help bioremediate As-contaminated sites [39]. PGPB contribute to cesium (Cs^+^) biosorption through exopolysaccharide production and biofilm formation in *Nocardiopsis* sp. Phosphate-solubilizing PGPB also promote metal precipitation; phosphatases release inorganic phosphate from organophosphates (e.g., glycerol 2-phosphate), which binds metal ions to form metal-phosphate deposits on biomass. Moreover, microbial-induced carbonate precipitation (MICP) effectively immobilizes metals by converting soluble ions such as Pb^2+^ and Cu^2+^ into insoluble carbonates, thereby reducing their bioavailability and toxicity [40].

### 3.4. Limitations of PGPB Application and Ecological Concerns

The effectiveness of PGPB inoculation has been known for centuries, but before it can be successfully utilized in agriculture, several problems must be resolved. The production of biochemical PGPB characteristics and activities is based on laboratory-based testing. Because the efficacy of PGPB formulations cannot yet be predicted from in vitro data, there is an imperative need to develop PGPB formulations tailored to field applications [41]. Another important factor is that PGPB can have different effects on plant growth at different times after their application. Thus, the effects of single (acute) and repeated (chronic) applications, as well as the object of the application, should be addressed. It is important to note that not all bacterial strains can survive long-term in soil, and it will be necessary to propagate bacteria in large numbers and ensure their bioactivity in the field [42].

Additionally, factors such as soil properties, plant species, and farming activities may influence the most abundant microbial taxa, despite their high survival rates. Soil temperature is one of the main causative factors of microbial performance. Some PGPB-produced polysaccharide-based biofilms protect symbiotic plant partners against environmental changes, such as changes in pH or temperature [27]. Soil nutrients can alter microbial communities and reduce the functionality of PGPB. The increasing overall diversity leads to complex relationships, reduces antagonism, increases food abundance, and decreases nutrient competition among microbes. However, this may hamper the mutualistic benefits of symbioses between the plant and PGPB. The viability of PGPB is determined by the production conditions (media, growth parameters), formulation (liquids, seed coatings, pellets), and transport stressors. It is difficult to develop inoculants that achieve high root colonization and persist in the soil. The best commercial strains must be quickly scalable in vitro, be bacterial-competent, be eco-friendly, coexist with the microbiota, and withstand abiotic stresses [43]. Large-scale culture of most PGPB isolates is difficult because they do not grow well in synthetic media compared with their natural habitats. These limitations may be overcome through genetic engineering, such as the use of genes encoding functional proteins that enhance PGPB colonization, stress tolerance, and the efficacy of growth promotion [44].

Moreover, genetically engineered PGPB can alter the native soil microbiota, making their regular colonization of host plants challenging. To overcome these challenges, non-thermal plasma (NTP) has emerged as a promising tool to enhance the viability and functional activity of PGPB. The fourth state of matter is plasma, a partially ionized gas composed of electrons, ions, and neutral species. Plasma, when used under different conditions, generates RONS that promote microbial metabolism, increase stress, and promote biofilm formation, among other effects. Low-dose plasma exposure in seed treatments enhances surface wettability, facilitates enzyme activity, and improves germination, thereby strengthening plant-microbe interactions and improving growth performance [45]. NTP has transformed agriculture by improving seed germination through surface decontamination, enhancing plant vigor, and inducing systemic resistance to biotic and abiotic stress. PTW that contains long-lived RONS is a powerful inactivator of various pathogens and provides nitrates as an efficient fertilizer [46]. These effects spread from microbial inactivation to activation, which is controlled by dosage and produces RONS. Its application to PGPB activation has rarely been explored. The viability and functional efficacy of isolated PGPB can be enhanced to achieve sustainable agriculture. Moreover, NTP treatment can modulate microbial activation through controlled exposure to RONS and may enhance stress adaptation and plant-beneficial functions. However, the NTP treatment also includes energy consumption, equipment cost, and field-scale scalability before practical agricultural application. Therefore, future approaches may involve integrating NTP with optimized carriers or microbial formulations to achieve stable and efficient PGPB delivery, as shown in Table 1.

## 4. Application of Plasma Treatment Systems in Agriculture

Many studies have reported that the potential of plasma technology to improve crop performance, enhance food safety, and promote sustainable production systems is increasingly recognized in agricultural research. Plasma produces a mixture of reactive oxygen and nitrogen species (RONS), including hydroxyl radicals, atomic oxygen, ozone, and nitrogen oxides. These reactive molecules can impair cellular integrity by triggering oxidative processes such as lipid peroxidation and protein modification [47,48,49]. Plasma treatments of seeds are broadly classified into direct and indirect methods. Such treatments are carried out using controlled plasma generation systems, including dielectric barrier discharge (DBD), corona discharge (CD), atmospheric pressure plasma (APP), and glow discharge (GD), to achieve reproducible physicochemical effects [50,51]. Plasma treatment processing conditions are precisely controlled by varying operational parameters, such as electrode configuration, power attributes (voltage, frequency, and waveform), gas flow rate, pressure, and temperature. DBD plasma treatment enhanced wheat germination potential compared with the control and improved plant height, root length, and fresh weight at the seedling stage [52].

**Figure 3:**
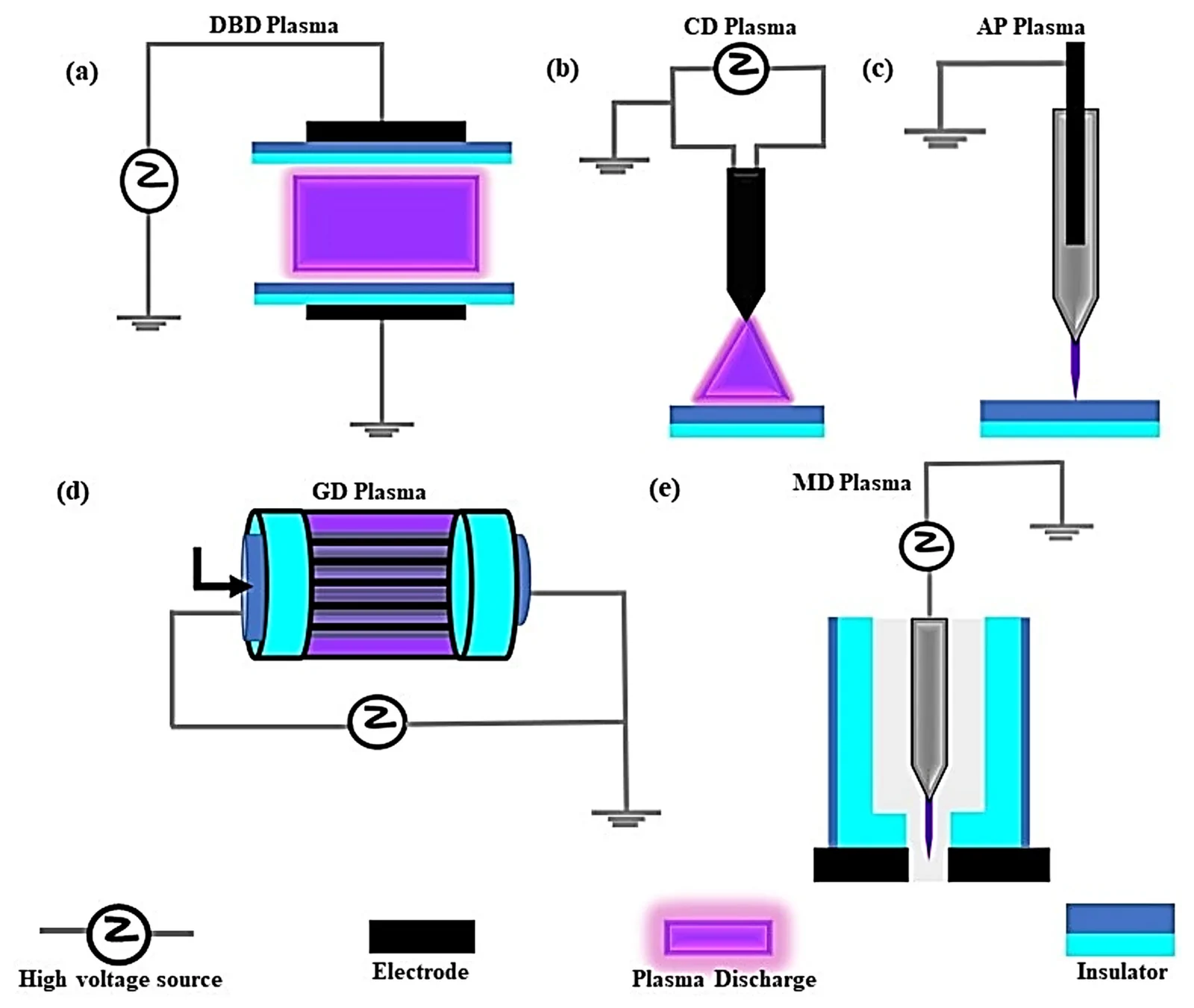
Schematic representation of plasma systems used in agriculture: (**a**) dielectric barrier discharge (DBD), (**b**) corona discharge (CD), (**c**) atmospheric pressure (AP), (**d**) glow discharge (GD), and (**e**) microwave discharge (MD) plasma.

CD plasma can improve nutrient uptake and alter seed surface properties, increasing water absorption. Its low energy consumption and straightforward system design make it appropriate for small-scale agricultural use. APP plasma operates at atmospheric pressure and does not require a vacuum infrastructure [49,53]. Moreover, GD plasma has been applied to treat seeds, enhance germination, and promote early seedling development. Exposure of *Raphanus sativus* L. seeds to low-pressure GD plasma at 20 Pa resulted in a significant increase in seedling length [52]. Furthermore, MD plasma treatment has been used in plant biology research to improve germination and seedling growth. Moreover, the MD plasma system has shown promise for degrading pesticides and other organic pollutants in water and soil. Their ability to generate dense plasmas containing reactive species puts them in a good position for use in various agricultural processes, such as seedling growth and environmental remediation [54]. These systems generate a diverse range of RONS, supporting their broad application in plant-related research (Figure 3). Plasma generation alters water chemistry and enhances seed germination and plant growth. Seed treatments mainly target surface microbial inhibition. 

Recent studies have found that RONS promote seed priming and contribute to microbial inhibition [55]. PTW has a high concentration of nitrate and nitrite, which can serve as nitrogen sources and are thought to be the main factors driving plant growth. Thus, the term “plasma fertilizer” was introduced. Additionally, one environmentally suitable alternative to nitrogen sources that mitigates the negative effects of chemical fertilizers is “plasma fertilizer” [56]. PTW positively impacts the growth and development of *Brassica rapa* (Chinese cabbage) plants, particularly when accompanied by magnesium (Mg^2+^) and zinc (Zn^2+^) ions. This treatment resulted in faster seed germination, stronger seedling development, higher protein and chlorophyll levels, and modulated gene expression, all of which led to stronger, healthier plants [57]. 

Plasma treatment also increased germination and seedling growth in lettuce. Short exposure times were more effective, while longer durations did not show additional benefits. PTW has a positive morphological impact and increases chlorophyll content, thereby increasing crop yield [58]. Furthermore, PTW depends on the chemical composition and treatment frequency, which modulate intracellular Ca^2+^ signaling inside the plant cell [59]. PTW treatment also affects seed testa and endosperm rupture, enhances gibberellin-3-oxidase activity, and upregulates expansin-A4 expression in Nicotiana tabacum [60]. The physicochemical characteristics of the soil are negligible after plasma treatment, but water retention improves. As a result, PTW could be a promising tool for improving plant access to soil moisture while having minimal impact on other soil characteristics [61].

### 4.1. Molecular Interaction of PTW for Growth and Development

**Figure 4:**
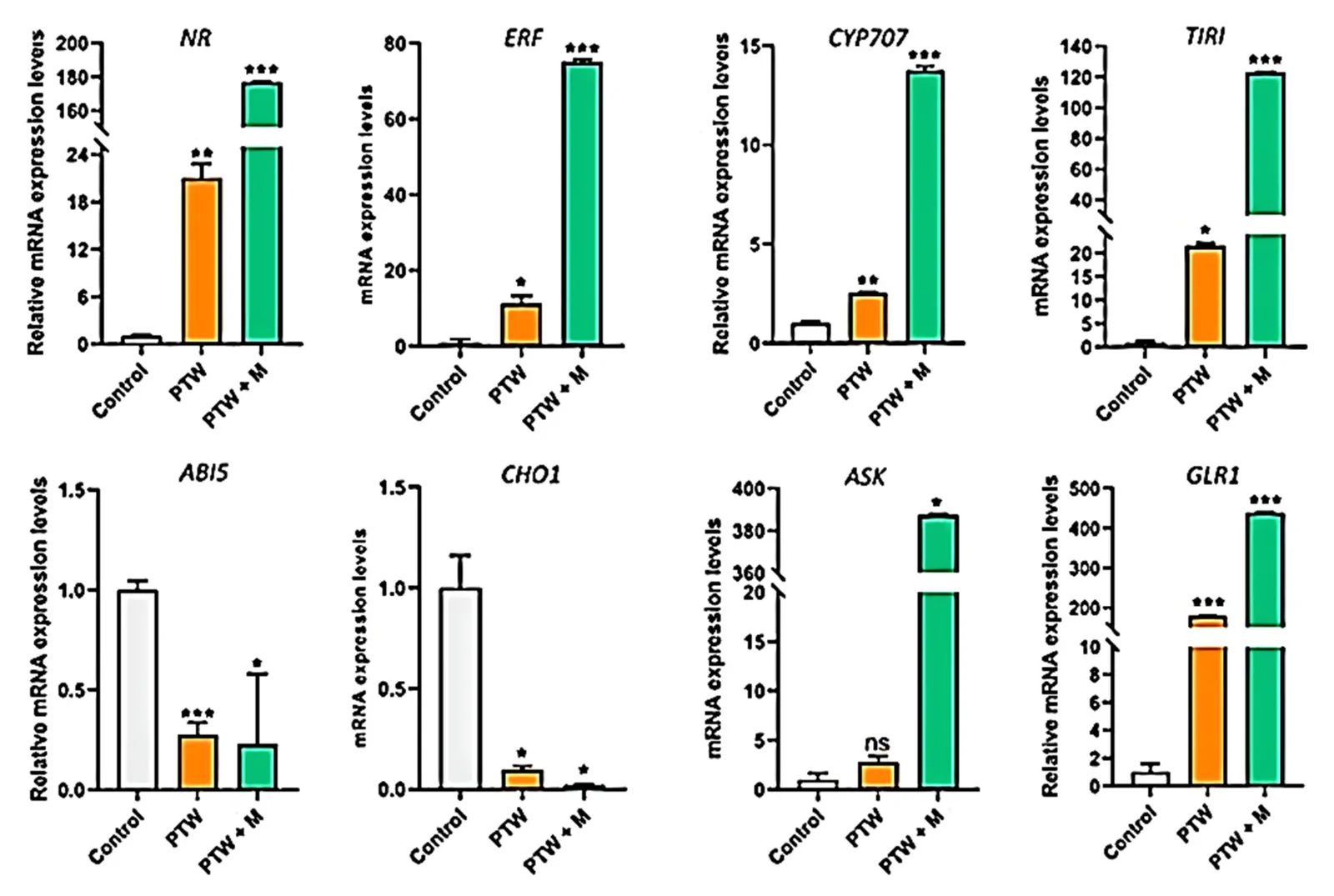
The molecular expression of *Pak Choi* seedlings after using plasma-treated water with metal ions (Mg^+2^ and Zn^+2^). Epigenetic gene expression analysis demonstrated that PTW RONS enhanced nitrate assimilation and plant growth genes, with physicochemical effects on Pak Choi seedlings. However, gene expressions are regulated by phytohormonal balance via auxin synthesis and abscisic acid catabolism, thereby leading to distinct stages of plant growth [57]. The statistical analysis of treatment groups is denoted by * *p* < 0.05, ** *p* < 0.01, and *** *p* < 0.001.

NTP technology has significant advantages in agriculture, including seed germination, plant growth, crop production, food preservation, and stress tolerance [62]. Plasma is also a significant source of nitrogenous compounds, including nitric oxide, nitrite, nitrate, dinitrogen trioxide, dinitrogen pentoxide, and ammonia [63]. These nitrogen species have high mobility and soil solubility and are more readily taken up by plants. As a result, they can be effectively used as sustainable natural fertilizers [64]. PTW containing Mg^2+^ and Zn^2+^ ions has a neutral pH, which enhances its effectiveness in stimulating Pak Choi growth. PTW with metal (Mg^2+^ and Zn^2+^) ions significantly increased seed germination, biomass accumulation, and leaf greenness, indicating better nitrogen assimilation through elevated chlorophyll and protein content. At the molecular level, PTW significantly upregulated nitrate reductase (*NR*) and ethylene response factor (*ERF*) genes and downregulated *ABI5* and *CHO1*, which are associated with growth inhibition. Moreover, PTW with Mg^2+^ and Zn^2+^ significantly increased the expression of *CYP707A2* and *TIR1*, which are involved in ABA catabolism and auxin biosynthesis. Consequently, PTW with Mg^2+^ and Zn^2+^ ions enhanced the growth and development of Chinese cabbage seedlings through RONS-mediated phytohormonal signaling (Figure 4) [57]. 

**Figure 5:**
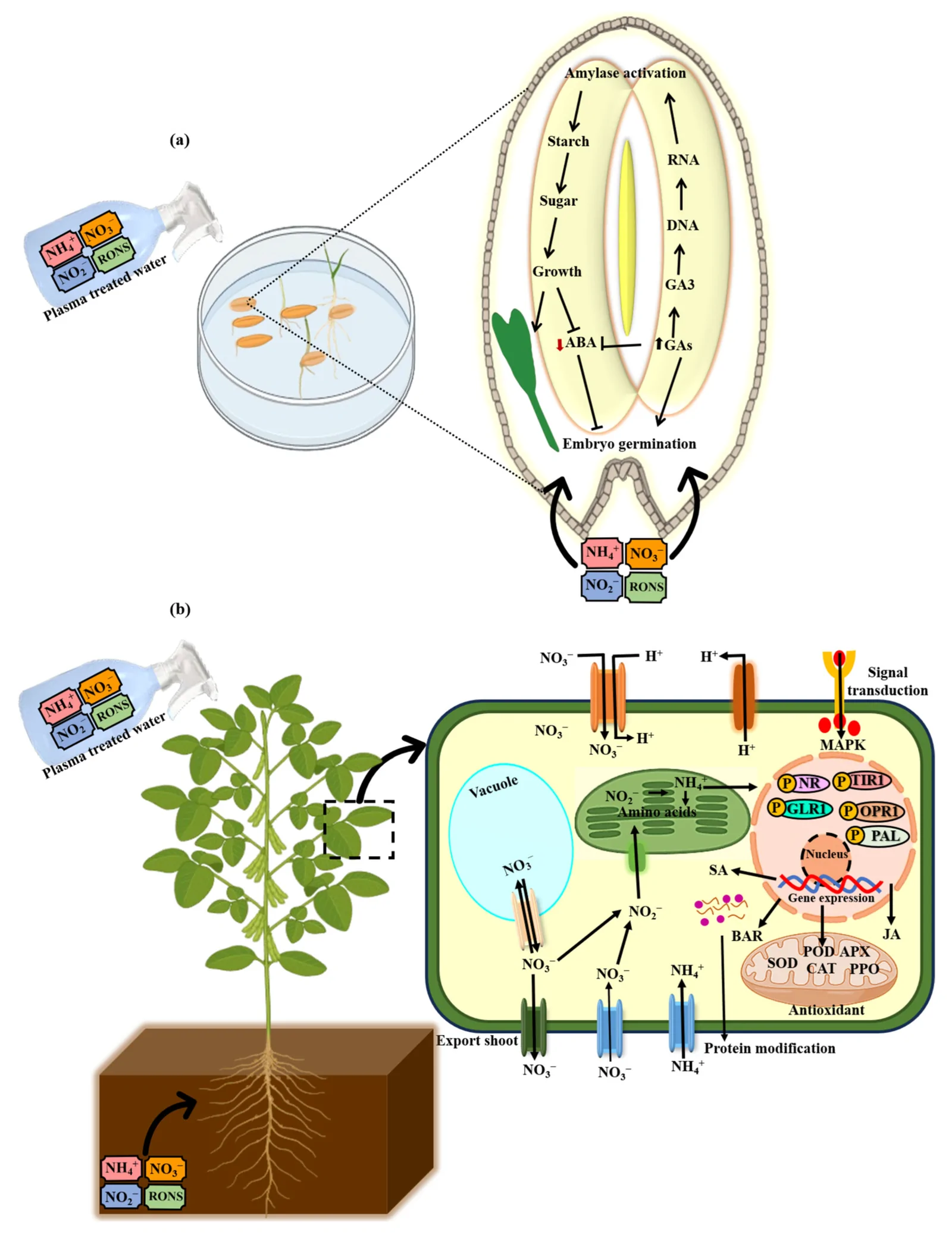
Molecular mechanisms underlying the influence of plasma-generated RONS at different growth stages. (**a**) PTW affects the seed testa and ruptures the endosperm, modulating hormonal balance as a signaling molecule to trigger germination. (**b**) PTW-derived nitrate (NO_3_^−^), nitrite (NO_2_^−^), and ammonium (NH_4_^+^) are taken up by roots via membrane transporters, reduced to NH_4_^+^, and incorporated into amino acids; excess nitrogen is either stored or transported to the shoot to support plant growth. Figure(s) partially created in BioRender. Javed, R. (2026) https://BioRender.com/sl1hbg2.

However, PTW may enable the use of plasma agriculture in pH-sensitive crops. Plant growth is influenced by soil pH, which significantly affects the availability of nutrients and minerals. Most plants function optimally at a neutral pH, which promotes ideal nutrient absorption and uptake [57]. Plasma treatment affects plant growth and development, including sterilization, enhanced germination, improved growth, and activation of defense mechanisms. RONS modulate major molecular pathways in seeds and seedlings, such as redox signaling, metabolic regulation, ABA catabolism, and gibberellin biosynthesis [65]. Molecular research on plasma treatment has targeted redox metabolism-regulating enzymes, such as peroxidase (POD), polyphenol oxidase (PPO), ascorbate peroxidase (APX), catalase (CAT), and SOD. The effects of plasma treatment on redox homeostasis have been reported across multiple plant species, including rice, radish, barley, and sunflower [66,67]. Plasma treatment activated redox enzymes, which subsequently regulate transcription, signaling, and downstream gene expression [68]. DBD plasma treatment has been demonstrated to improve seed germination and plant growth in maize by triggering antioxidant defense mechanisms. DBD plasma generated using an argon/air (Ar/air) mixture increased ROS-related enzyme activities in seeds and seedlings, accompanied by sustained upregulation of CAT and SOD expression. The enhanced activity of antioxidant enzymes subsequently promoted seed germination and early plant development [69]. The molecular investigation of plasma treatment has involved not only seed germination but also the stimulation of plant defense-responsive mechanisms, predominantly mediated by RONS (Figure 5). 

PTW induces MAPK signaling in tomato seedlings, leading to the expression of defense genes such as chitinase and β-1,3-glucanase. Furthermore, PTW enhanced the gene expression of OPR1, AOS, and PAL, involved in salicylic acid and jasmonic acid production, thereby boosting hormone-mediated defense pathways [68]. However, plasma treatment positively influences chlorophyll accumulation and net photosynthetic rate in wheat grown under drought stress [70]. Plasma treatment may modulate the dynamics of photosynthetic pigments and photophosphorylation processes. The higher chlorophyll content found in plants treated with plasma is likely associated with enhanced photosynthetic efficiency and physiological activity. Furthermore, stomatal conductance and chlorophyll concentration are significantly associated, indicating greater capacity for CO_2_ uptake and assimilation [71]. The effect of NTP treatment depends on plasma types and plant species, which induce alterations at the biochemical, transcriptional, and metabolite levels in plants under abiotic stress (Table 2). 

### 4.2. Plasma Basis of Adaptive Signaling to Abiotic Stress

#### 4.2.1. Molecular Signaling Pathways of Abiotic Stress by Plasma Technology

Plants undergoing severe or persistent stress must divert energy from growth and reproduction to stress perception, signaling, and defense responses, which eventually lowers plant metabolism and adaptability. On the other hand, low-intensity stress may cause no discernible loss in healthy plant performance because it can activate defensive mechanisms without imposing excessive energetic expenditures [90]. Mild stress exposure can induce long-lasting beneficial effects in plants. Mild stress activates and modifies stress-responsive signaling networks, thereby preparing the organism for subsequent stress events. As a result, primed plants typically display faster, stronger, and more efficient responses to more severe stress than non-primed plants [91].

NTP-treated CBZ solutions have improved germination and seedling growth, suggesting a mitigation of CBZ toxicity. This study provided insights into enhancing wastewater treatment technologies in the agricultural sector and addressing pharmaceutical contamination in aquatic habitats [92]. Plasma treatment promoted root growth under zinc oxide nanoparticle stress in pepper (*Capsicum annuum* cv. *cayenne*). Plasma priming increased carotenoids, chlorophyll a, and soluble phenolic compounds, thereby enhancing the expression of peroxidase and phenylalanine ammonia-lyase (PAL) [93]. Plasma treatment increased peroxidase activity, improved resistance to multiple abiotic stressors by enhancing membrane integrity, and reduced electrolyte leakage. When cotton seedlings were exposed to chilling stress and reduced water availability, plasma seed treatment significantly enhanced germination efficiency and water uptake [94]. All these results suggest that NTP is an effective priming method for a variety of environmental challenges that plants frequently face. Importantly, optimizing the NTP dose typically stimulate growth and, more prominently, induces adaptive responses that mitigate stress-induced damage. 

#### 4.2.2. Plasma Triggers Plant Tolerance Against Salinity Stress

Many studies have reported that NTP treatments can enhance salinity tolerance in cereal crops such as rice and wheat. In wheat seedlings, DBD plasma increased salt stress resistance by inducing HSFA4A expression and elevating peroxidase and phenylalanine ammonia-lyase activities, thereby activating defense responses and alleviating salinity-induced damage [82]. However, PTW as a nutrient solution noticeably enhanced shoot and root elongation, biomass accumulation (up to 80.5%), and chlorophyll content in crops such as Bok choy under NaCl stress (20–100 mM). Salinity-responsive genes, such as *WRKY2*, *HHP3*, and *ABI1*, were significantly upregulated in plants grown with plasma-treated Hoagland solution in tandem with this growth enhancement. However, these findings indicate that plasma activation of nutrient solutions in hydroponic systems can substantially improve plant growth, physiological performance, and salinity stress tolerance [95]. 

**Figure 6:**
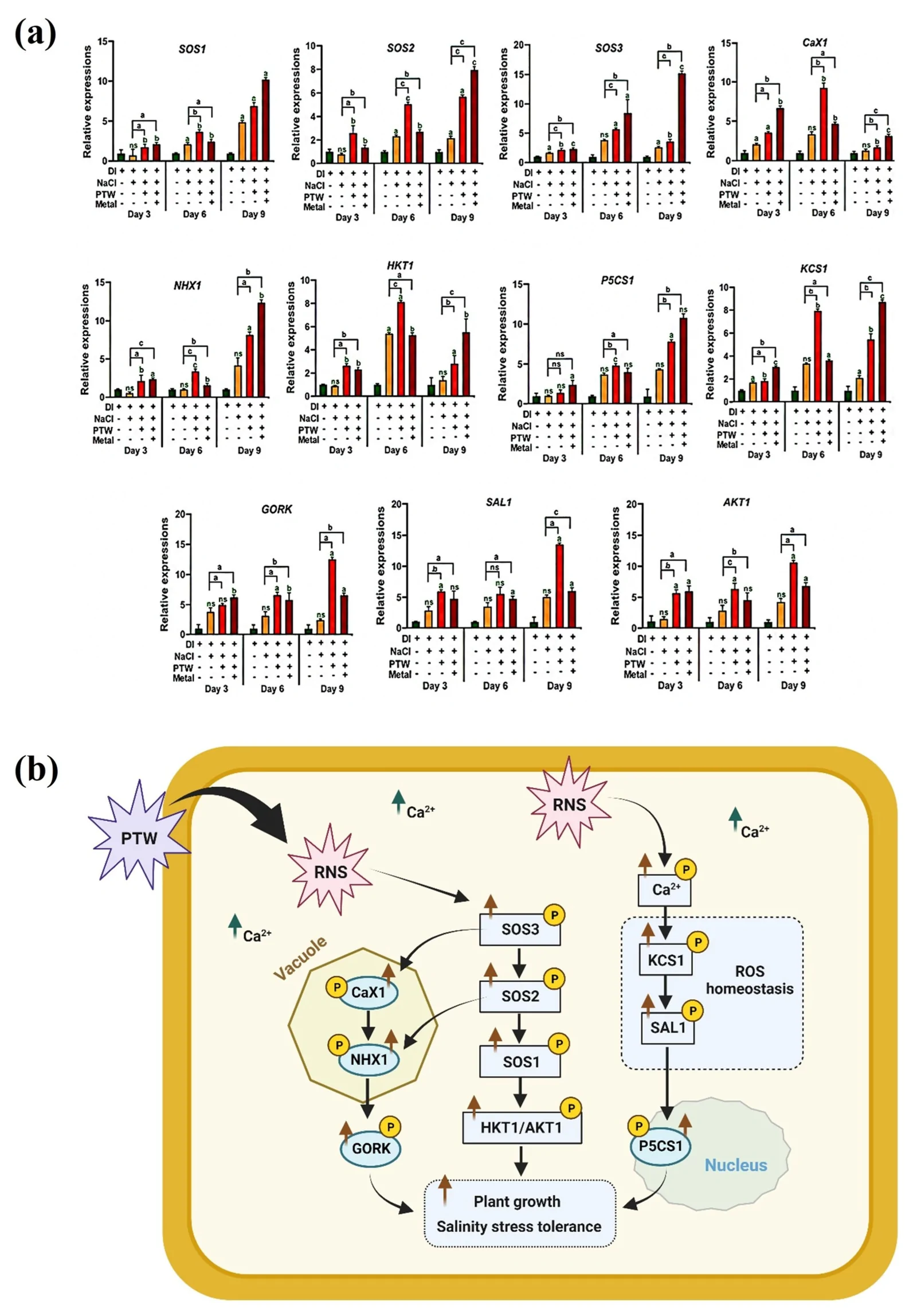
(**a**) Gene expression of Pak Choi seedlings treated with PTW with metal ions (PTW + M) under salinity stress. (**b**) Irrigation with PTW and PTW + M significantly affected the molecular mechanisms associated with the salt overly sensitive (SOS) pathway in Pak Choi seedlings under salinity stress. The genes implicated in the regulation of osmotic and ionic balance were activated because of the plasma treatments. PTW and PTW + M increased levels of cell-signaling molecules, such as calcium ions (Ca^2+^) and nitric oxide (NO), as these molecules are known to play a combined role in regulating stress-tolerance responses [83].

The effectiveness of plasma seed priming as a technique to increase early-stage salt tolerance and promote *P. koelziana* establishment under salinity stress improved seedling performance in both moderate and severe salinity [80]. Furthermore, PTW with metal ions (PTW + M) significantly improved the growth performance of Pak Choi seedlings exposed to 100 mM NaCl, as observed on days 3, 6, and 9. PTW, enriched in RNS, predominantly regulated the transcription of salt-overly sensitive (SOS) pathway genes, including *SOS1*, *SOS2*, and *SOS3*, thereby enhancing cytosolic Ca^2+^-binding activity. Both PTW and PTW + M contained mixed RNS (NO, NO_2_^−^, and NO_3_^−^) together with Mg^2+^ and Zn^2+^ ions, which collectively upregulated genes associated with ionic homeostasis and redox regulation, such as *CaX1*, *NHX1*, and *HKT1*. In addition, the expression of membrane potential–related genes (*KCS1*, *GORK*, *SAL1*, and *AKT1*) was enhanced, thereby improving growth under saline conditions (Figure 6a,b) [83]. 

Overall, PTW emerges as a promising priming agent for enhancing plant tolerance to environmental stress, as optimized doses promote growth and immune regulation, thereby triggering adaptive responses that alleviate stress-induced damage. 

#### 4.2.3. Plasma Triggers Plant Tolerance Against Drought Stress

NTP pre-treatment improved seedling growth and induced tolerance to drought stress. This increased tolerance was associated with greater accumulation of osmo-protectants, including proline and soluble sugars, and upregulation of key stress-responsive genes, such as *SnRK2* and *P5CS*. In addition, NTP priming reduced oxidative damage, as indicated by lower reactive oxygen species levels and lipid peroxidation, while stimulating antioxidant enzyme activity and abscisic acid accumulation, thereby strengthening stress-adaptive responses [74]. Moreover, NTP priming in tomato mitigated polyethylene glycol (PEG)-induced drought stress, emphasizing the signaling roles of H_2_O_2_ and NO in plant defense. After NTP treatment, similar H_2_O_2_ and NO-mediated responses were observed in barley seeds. The effects of plasma exposure were assessed in *Brassica napus* L. under drought stress in both drought-sensitive and tolerant cultivars. Plasma-treated seeds from both genotypes exhibited reduced oxidative damage and maintained metabolic balance. These responses were closely associated with enhanced antioxidant defense, particularly superoxide dismutase and catalase [72].

#### 4.2.4. Plasma Triggers Stress Tolerance Against Heavy Metal Stress

Soil has become heavily polluted with heavy metals due to intensive anthropogenic activities such as mining and the overuse of inorganic fertilizers and pesticides. The accumulation of toxic metals adversely interferes with seed germination and key developmental processes in plants, ultimately impairing growth, yield, and crop quality (Iqbal et al., 2025) [92]. Cadmium stress increased hydrogen peroxide accumulation and increased the expression of the cadmium transporter genes *TaLCT1* and *TaHMA2*. Seed pre-treatment with NTP mitigated these effects by enhancing the activity of antioxidant enzymes, including catalase and superoxide dismutase. Additionally, NTP reduced cadmium accumulation by suppressing the expression of the cadmium transporter gene. Plasma treatment increased the expression of wheat antioxidant genes, such as TaSOD and TaCAT, thereby substantially reducing cadmium-induced oxidative damage. The decline in cadmium accumulation has been correlated with reactive species produced by plasma, which decreased cellular pH and limited metal permeability in the rhizosphere. While increasing total soluble protein, plasma treatment also reduced electrolyte leakage and cell death in roots and shoots [87].

**Figure 7:**
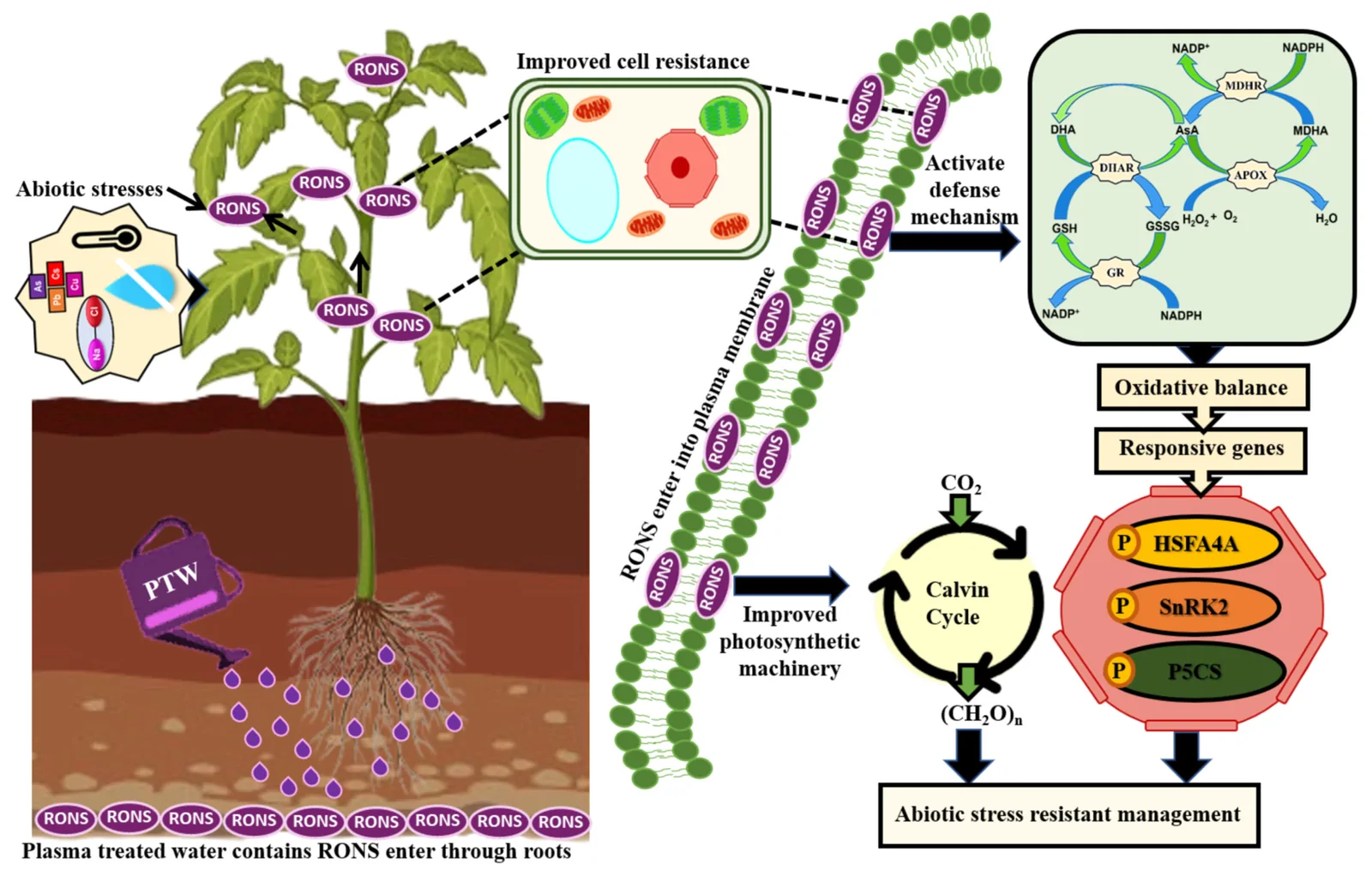
Molecular mechanisms and transport pathways of PTW inside plant cells against various abiotic stresses. PTW generates reactive oxygen and nitrogen species (RONS), which act as redox signaling molecules, not only reducing the negative effects of stress but also enhancing antioxidant enzyme activity, stress-response gene expression, and metabolic activity. Figure(s) partially created in BioRender. Javed, R. (2026) https://BioRender.com/c1dxcpe.

Moreover, PTW reduced catalase (CAT) activity, indicating more efficient detoxification of hydrogen peroxide under arsenic stress. Alternatively, the enzymatic activity of Guaiacol peroxidase (GPOX) increased markedly in roots exposed to PTW priming, thereby promoting redox homeostasis and protecting the photosynthetic machinery [96]. The molecular representation of PTW inside the plant cell under various abiotic stresses is shown in Figure 7. 

However, metal and metal oxide nanoparticles have beneficial effects on plants, such as remediating heavy metal contamination, even though numerous studies have shown that they may be cytotoxic to plant cellular systems, particularly at high concentrations [97]. Another study has shown that pretreatment with NTP promotes bell pepper development and protects against the toxicity of zinc oxide (ZnO) nanoparticles. These effects have been attributed to reactive species produced by plasma, such as nitric oxide, ozone, and UV light, which increase the activity of antioxidant enzymes such as phenylalanine ammonia lyase and peroxidase, hence reducing the toxicity caused by nano-ZnO. Similarly, *Melissa officinalis* seedlings treated with plasma improved resistance to high concentrations of ZnO and selenium nanoparticles [98]. The phytotoxic effects of high concentrations of selenium nanoparticles were effectively mitigated in *Cichorium intybus* seedlings before plasma treatment, thereby significantly improving seedling growth and development [99]. 

## 5. Plasma-Induced Metabolic Reprogramming in Plant Microbiomes

**Figure 8:**
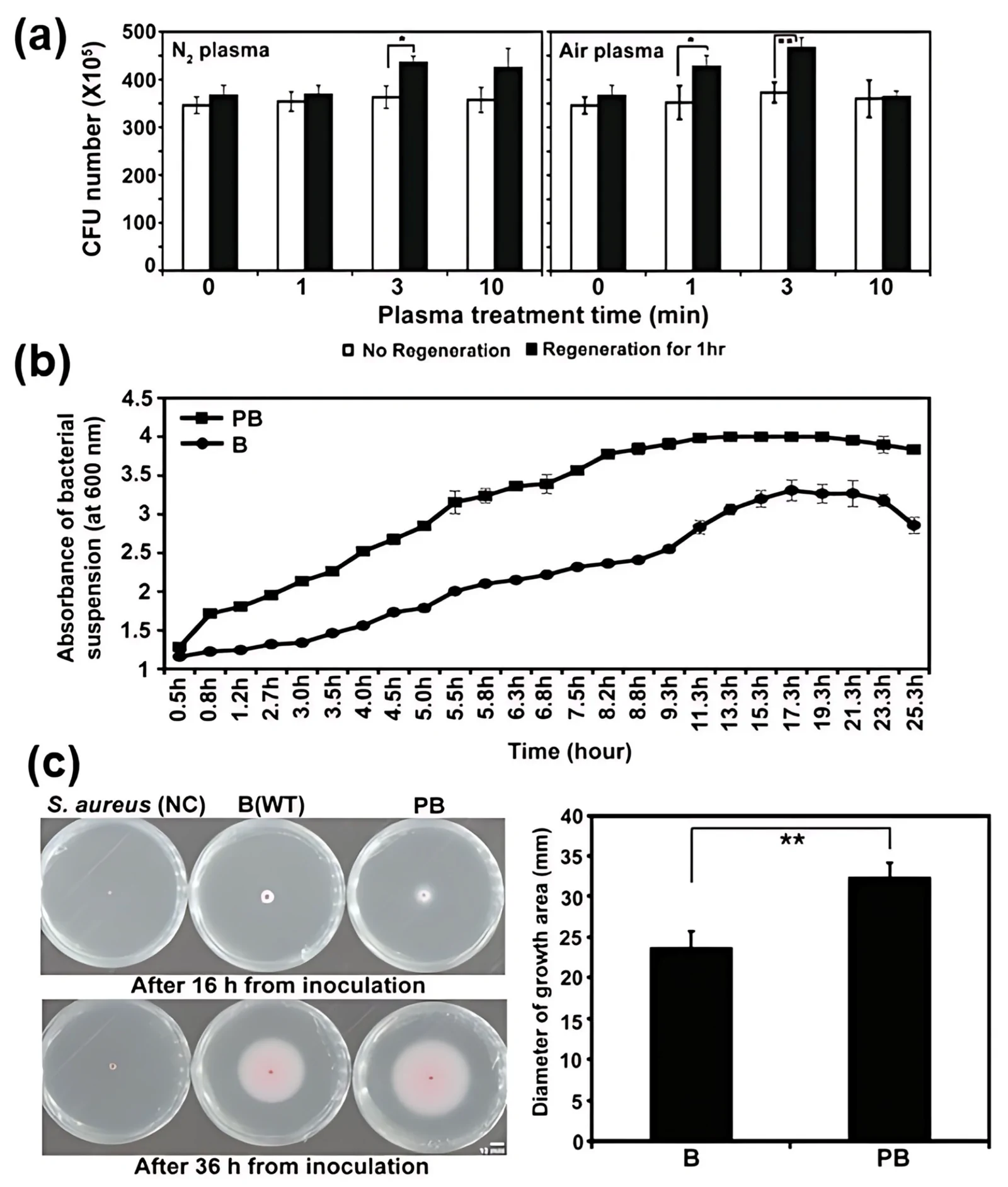
Illustration of Bacterial vitality after plasma treatment: (**a**) Colony-forming units (CFUs) of bacteria after exposure to N_2_ and air plasma, (**b**) bacterial growth assessed during incubation by measuring optical density at 600 nm, and (**c**) swarming assay of bacteria on LB medium and the respective swarm-zone diameter [45]. The statistical analysis of all measurements is denoted by * *p* < 0.05, ** *p* < 0.01, and *** *p* < 0.001.

NTP technology generates a physicochemical mixture of ionized gases and has become an innovative tool in agricultural research for regulating plant microbial communities and may improve plant metabolism [100]. Plant microbiomes are complex, dynamic communities of beneficial microorganisms essential for regulating plant growth and development and mediating plant responses to interactions with both biotic and abiotic environmental factors [101]. Moreover, microbial communities have been exposed to various environmental stressors, and their structure and functional processes are specific to root colonization [102]. Recent reports show that plasma treatments can significantly modify microbiomes, either through direct action of plasma-derived RONS on microbial cells or indirectly via plasma-induced alterations in the physicochemical characteristics of the plant surface [101,103]. The microbial composition may alter after plasma exposure, which induces secondary metabolite production, thereby affecting plant growth, defense, and stress tolerance. The future of plasma agriculture needs to understand how these microbiome changes affect plant metabolism. Although plasma technology has advantages, overexposure can disrupt plant physiology and reduce yield by 15–30%. Nevertheless, it is a promising weed management tool because it can destroy weed tissue and sterilize soil surfaces [104]. Moreover, recent developments in plasma technology enable modulation of plant microbiomes. NTP treatments without heat may also be used to increase microbial activity, thereby improving plant growth and stress resistance, especially in rice (Figure 8) [45]. 

Furthermore, plasma treatments significantly alter plant-microbe interactions by facilitating the establishment of microbial communities, enhancing pathogen tolerance, and elevating phytohormone levels [105]. Plasma treatment controls the microbiome by generating reactive species that selectively affect beneficial and harmful microorganisms in different ways. Such a selective response arises from distinct biological and structural peculiarities that determine microbial sensitivity to plasma-derived agents [106]. The beneficial soil microorganisms induced stress tolerance through efficient antioxidant defense and DNA repair. Consequently, favorable rhizobacteria may be preserved at approximately 60–70% viability under conditions that eliminate up to 90% of pathogenic microbes [53]. However, air plasma treatment influences specific plant microbiomes, effectively reducing microbial loads without compromising crop quality [107]. Similarly, air plasma reduces water consumption during lettuce cleaning and effectively alters surface microbial communities [108].

### 5.1. Effects of Plasma on Beneficial Microbes to Combat Stress

Plasma treatment stimulates beneficial soil microbes, promoting the growth of specific PGPB and inhibiting plant pathogens. Plasma technology is an alternative, eco-friendly method for improving plant health by reducing reliance on synthetic pesticides [109]. The application of innovative techniques, such as pulsed-discharge mode and intelligent control systems, may increase energy conservation in plasma technology, making it a practical alternative to conventional chemical treatments and reducing initial power requirements. Furthermore, the growth of PGPB and *Klebsiella pneumoniae* was significantly stimulated by plasma exposure without reducing cell membrane integrity [110].

### 5.2. Role of Plasmid-Mediated Plant-Beneficial Microbiome Engineering

Plasma treatment significantly alters the plant microbiome and may modify the plasmid content of associated microbes. Since plasmids regulate essential characteristics, including antimicrobial resistance, pathogenicity, and positive microbial activity, research into plasmid-induced alterations in the plasmidome is of particular interest. The impact of plasma treatment on both short-term microbial reactions and long-term plasmid-based adaptation is essential for the development of sustainable agricultural processes [111]. Recent research on plant microbiomes has identified an increasing trend in plasmid-associated resistance genes. Animal-based fertilizers and contaminated water may significantly change plant-beneficial microbiota and facilitate horizontal gene transfer. Although plasmids are disseminated through cross-contamination and manure application, their interactions within soil microbiomes remain poorly understood. Plant microbial communities significantly impact growth and development, driving increased interest in plant metagenomics and plant growth-promoting microbes. The presence of plasmid traits in these microbiomes increases interactions between plants and microbiomes by providing adaptive benefits under unfavorable environments. Since abiotic stress is a significant constraint on plant productivity, microbial diversity-enhancing mechanisms, such as enhanced nutrient uptake and plasmid-mediated phytohormone production, are important in alleviating stress while supporting microbial diversity [102]. Moreover, plasmids are mobile genetic elements that participate in the functional adaptation of rhizosphere microbial communities through horizontal gene transfer (HGT). Plasmids frequently carry genes involved in plant growth promotion, such as nitrogen fixation, phosphate solubilization, siderophore biosynthesis, phytohormone production, and resistance to abiotic stresses such as drought, salinity, and heavy metal toxicity. Furthermore, plasmids also act as metal resistance determinants, such as copper (Cu), chromium (Cr), and cadmium (Cd) detoxification, which accelerate microbial survival under heavy metal stress and may contribute to phytoremediation processes [112]. Although plasma-generated RONS induce microbial physiology, DNA stability, and stress responses, their impact on plasmid stability and HGT within rhizosphere communities remains poorly understood. Future studies integrating plasmidomics, metagenomics, and functional analyses are required to elucidate how plasma-driven microbial genetic exchange affects ecosystem stability, beneficial microbial traits, and biosafety considerations.

The genes on the plasmids encode important microbial functions, such as motility, adhesion, and biofilm formation, that help plants form specific microbial communities. These plasmids also encode pollutant-degradation pathways, such as those for phenol and toluene, in the rhizosphere. Recent studies demonstrate the significance of the plasmidome in regulating plant responses to abiotic stress, including plasma treatment. Plasma treatment induces oxidative stress, which triggers a cascade of physiological and molecular responses in plant cells. The plant microbiome comprises diverse microbial communities, with plasmid-mediated functions that regulate plant health and stress tolerance. It has been indicated that plasma treatment can alter the composition and activity of the microbiome, thereby enhancing plants’ resilience to environmental stressors [113,114].

### 5.3. Plasma-Induced Transcriptional Reprogramming in Plant Microbiomes

Plasma triggered a cascade of transcriptional alterations as microbes activated defenses, counteract damage, and acclimate to stressful conditions. Cutting-edge transcriptomic methods, such as RNA sequencing (RNA-seq), have shown that plasma exposure significantly alters the gene expression associated with oxidative stress responses, metabolism, and DNA repair pathways [115,116]. The microbial genes encoding ROS-detoxifying enzymes are consistently upregulated after plasma treatment. These enzymes suppress the effects of ROS, thereby defending cells against oxidative damage during stress responses. Consequently, the plant’s adaptive responses can be improved by intensive microbes through microbial adaptation to post-plasma exposure [115]. On the other hand, plasma treatment influenced the metabolic activities of microbial communities. Studies of transcriptional profiling have shown that plasma induced downregulation of genes associated with major metabolic pathways, including oxidative phosphorylation, glycolysis, and the tricarboxylic acid (TCA) cycle [115]. The low levels of central metabolic pathways indicated a slowdown in energy conservation, as the cells prioritized survival during oxidative stress over replication. The inhibition of metabolic pathways could lead to insufficient ATP production, a slower growth rate, and a more energy-efficient phenotype, thereby enabling microbes to withstand unfavorable conditions [115,117]. Plasma treatment alters gene expression in microbes, paving the way for targeted applications. Plasma beneficially improved the stress tolerance of culturable microbes, enabling them to defend crops better [118]. 

### 5.4. Plasma-Induced Metabolic Shift in Plant Microbiomes

NTPs generate RONS, which interact with plant tissues, leading to alterations in primary and secondary metabolites [48]. The applications of metabolite changes span diverse fields, with the most prominent being agriculture, where plasma technology has the potential to enhance plant resistance to abiotic stress. One of the most widely studied results of plasma treatment is the control of antioxidant metabolites, such as ascorbic acid, flavonoids, and phenolic acids [119,120]. Moreover, the plasma treatment generates metabolites that elicit a robust defense strategy. The system eliminates ROS and enhances cellular stability, thereby reducing oxidative damage. Plants are more tolerant of abiotic stress as their antioxidant levels increase, thereby improving their growth and performance in such adverse environments [121]. Plasma treatment also enhanced nitrogen-containing metabolites such as glutamine and proline, transforming nitrogen metabolism. This stress-responsive reallocation emphasizes this adaptive reallocation. The metabolism of carbohydrates is also affected by plasma [83,122]. Plasma is intricately involved in metabolomic reprogramming as part of a multifaceted oxidative stress response that affects primary and secondary metabolism. Adaptations in plant cells following plasma exposure can enhance resilience to abiotic stress, underscoring plasma’s potential to improve crop vigor and productivity.

## 6. Conclusions and Future Prospects

In summary, the above-reviewed studies demonstrated that abiotic stresses severely impair plant production and yield by disrupting physiological processes such as photosynthesis, water balance, and oxidative homeostasis, thereby diverting energy from growth to survival mechanisms. Secondly, PGPB-mediated stress management may improve plant resistance to abiotic stress via ACC deaminase activity, regulation of phytohormones, and increased nutrient solubilization. Nevertheless, they are not usually effective in field applications, where their effectiveness is reduced by low survival, non-uniform root colonization, and environmental fluctuations under stress. Thirdly, NTP technology is a more integrative, complementary approach. Plasma-generated RONS serve as signaling molecules, triggering stress-responsive pathways to prepare plants to be more tolerant. At the same time, plasma treatment stimulates PGPB activation, root colonization aptitude, hormonal balance, and overall crop performance. This review has revealed the potential of plasma technology in agriculture, particularly for plant-microbe interactions, and presents a promising avenue for enhancing plant health and productivity under abiotic stress. Plasma treatments have significant potential in promoting PGPB activity, and the mechanism by which plasma induces RONS accumulation in plants remains to be elucidated. These benefits contribute to a more sustainable, eco-friendly approach to agriculture, reducing reliance on chemical pesticides and fertilizers. The application of plasma microbe-mediated stress mitigation significantly enhances stress tolerance in plants by improving photosynthetic efficiency, cellular antioxidant levels, nutrient uptake, and the regulation of biomolecular mechanisms. Plasma treatment can provide a cost-efficient and environmentally friendly approach to improving abiotic stress tolerance in plants by delivering essential nutrients. Consequently, crops can yield more biomass or fruit, even in challenging environments, helping mitigate the severe alterations to the world’s climate.

Further studies are required to investigate the interactions between plasma and plant microbiomes and to assess the adverse effects of residual plasma treatments under abiotic stress. The transition from the laboratory to the field is incredibly challenging without promising results from field trials. Plants are often exposed to multiple abiotic stresses rather than individual abiotic stresses, such as drought combined with salinity or heavy metal contamination, resulting in more complex physiological and molecular responses in agricultural fields. Although numerous studies have demonstrated the beneficial effects of NTP-PGPB in individual stress conditions, their multiple stresses remain unexplored. Likely, the effectiveness of NTP-PGPB may also be influenced by multiple signaling pathways, the durability of RONS, and microbial colonization under combined stresses. Therefore, future studies should investigate the efficacy and mechanisms of NTP-PGPB treatments under combined abiotic stress conditions to enable agricultural applications.

Future studies are considered necessary before starting field applications, such as assessing the long-term effectiveness of post-plasma treatment under field conditions and the impact of plasma treatment on the plant–microbial interaction transcriptome, proteome, and metabolome. An efficient and eco-friendly method is urgently required to achieve more sustainable agricultural production. Furthermore, the integration of plasma treatment can provide valuable insights into the interactions of plant microbial communities and stress mitigation in large-scale applications. The combined approach represents a paradigm shift from single-factor interventions to system-level modulation of plant-microbe interactions under unfavorable environments. Overall, this combined approach represents a paradigm shift: single-factor interventions are no longer used; rather, modulating plant–microbe interactions at the systems level under stress remains a fundamental approach. The combined application of plasma-mediated PGPB creates a multifactorial, environmentally sustainable approach to crop productivity amid increasing climate variability. Nevertheless, effective translational outcomes following plasma exposure will require mechanistic understanding, parameter optimization, and rigorous validation across a variety of agroecosystems. 

## Figures and Tables

**Table 1:** Comparison of different treatment strategies for improving PGPB-efficient application.

Treatment	Advantages	Limitations
Microbial encapsulation	Protects from desiccation and UV exposure, improves shelf life, and enables target delivery	Cost-effective, biodegradability, large scalability challenges
Carrier optimization	Increase survival rate, improved root colonization with nutrient uptake	Environment-dependent, which reduces the universal probability
Gene-edited PGPB	Improvement of targeted genes and beneficial traits	Metabolic impairment, public acceptance, ecological risk
NTP priming	Non-chemical, microbial stimulation, rapid modification, and eco-friendly	High energy input, limited scalability validation

**Table 2:** The effect of plasma treatment on stress resilience in different plant species under abiotic stress.

Plant Species, Plasma Types	Main Effects	Ref.
**Drought stress**		
Oilseed rape, RF discharge	Improved germination and increased antioxidant defenses, reducing lipid peroxidation under drought (15% PEG-6000).	[72]
Tomato, Plasma Jet	Enhanced pathogen resistance and stimulated hormone-dependent signaling gene expression.	[73]
Wheat (*Triticum aestivum* L.), DBD	Increased cellular water retention by improving soluble sugar (16.4%) and proline (12.7%) levels, thereby further enhancing the expression of stress-responsive genes, such as *LEA1*, *SnRK2*, and *P5CS*.	[74]
Licorice seeds (Glycyrrhiza), DBD, and SDBD	Improved germination rate, seedling growth, and development, which increased antioxidant enzymes (catalase and ascorbate peroxidase).	[75]
*Poa pratensis* L. Plasma reactor	Enhanced photosynthetic performance, osmolyte accumulation, and antioxidant enzyme activities.	[76]
*Avena sativa* L. DBD (air, argon, and 5 kV, 30 s)	Enhanced early seedling growth, photosynthetic efficiency, and soluble protein levels, and decreased malondialdehyde accumulation.	[77]
Alfalfa (*Medicago sativa* L.), RF discharge	Improved seed germination and vigor index and induced drought tolerance, only when used within a certain power range.	[78]
**Salinity stress**		
Barley (*Hordeum vulgare* L.), DBD reactor	Plasma treatment increased glutathione and carotenoid levels, which scavenge excess reactive oxygen species and minimize oxidative stress-induced damage.	[79]
*Prosopis koelziana*, DBD	Plasma improved seed germination from 44% and 21% to 100% and 68% under 100 mM and 200 mM NaCl, respectively.	[80]
Rice (*Oryza sativa*), NTP (10 kV), SA (2 mM)	Seed priming improved germination and nutrient uptake in coastal or irrigated fields under salinity (150 mM NaCl).	[81]
Wheat (*Triticum aestivum*), DBD	Plasma treatment increased heat shock transcription factor-4A gene expression and peroxidase and phenylalanine ammonia-lyase activities by 25% and 21%, respectively.	[82]
Pak Choi (*Brassica rapa* subsp. *Chinensis* L.), ME-CDBD	PTW and PTW + M treatments noticeably improved plant biomass up to 54.4%, shoot length by 18.1%, and root length by 26.0% relative to the control.	[83]
Saffron (*Crocus sativus* L.), DBD	Plasma increased corm mass, photosynthetic pigment, and protein content.	[84]
Wheat (*Triticum aestivum*), RF plasma reactor	NTP seed priming substantially enhanced nutrient acquisition with nitrogen and potassium uptake under moderate salinity and improved phosphorus and potassium uptake under high salinity stress.	[85]
**Heavy Metal stress**		
Water spinach, Plasma Jet	Plasma treatment decreased the bioconcentration factor of cadmium accumulation from 0.864 to 0.54, but it had no noticeable impact on lead uptake and crop production.	[86]
Wheat (*Triticum aestivum* L.), LPDBD (Ar/O_2_)	LPDBD plasma seed treatment decreased cadmium bioavailability and alleviated cadmium-induced growth suppression.	[87]
Soybeans (*Glycine max*)	Plasma treatment boosted soybean germination and led to a five-fold reduction in lead accumulation.	[88]
*Arabidopsis thaliana*, DBD	Seeds irrigated with zinc-tainted water and treated with oxygen plasma for 30 min exhibited the highest height and growth and showed reduced toxicity.	[89]

## Data Availability

All data supporting the findings of this study are provided in the main manuscript.
